# 2-Year-Old and 3-Year-Old Italian ALS Patients with Novel *ALS2* Mutations: Identification of Key Metabolites in Their Serum and Plasma

**DOI:** 10.3390/metabo12020174

**Published:** 2022-02-12

**Authors:** Mukesh Gautam, Renata Del Carratore, Benjamin Helmold, Alessandra Tessa, Oge Gozutok, Navdeep Chandel, Halil Idrisoglu, Paolo Bongioanni, Roberta Battini, P.Hande Ozdinler

**Affiliations:** 1 Department of Neurology, Feinberg School of Medicine, Northwestern University, 303 E. Chicago Ave, Chicago, IL 60611, USA; mukesh.gautam@northwestern.edu (M.G.); BenHelmold2023@u.northwestern.edu (B.H.); oge.gozutok@northwestern.edu (O.G.); 2 Clinical Physiology Institute of National Council of Research, Via Moruzzi 1, 56100 Pisa, Italy; rdc@ifc.cnr.it; 3 IRCCS Stella Maris Foundation, Calambrone, 56100 Pisa, Italy; alessandra.tessa@fsm.unipi.it; 4 Department of Medicine, Biochemistry and Molecular Genetics, Northwestern University Feinberg School of Medicine, Chicago, IL 60611, USA; nav@northwestern.edu; 5 Department of Neurology, Istanbul University, Istanbul 34116, Turkey; haidris@istanbul.edu.tr; 6 Spinal Cord Injuries Section, Department of Medical Specialties, Azienda Ospedaliero-Universitaria Pisana, Via Paradisa 2, 56100 Pisa, Italy; p.bongioanni@AO-pisa.toscana.it; 7 NeuroCare onlus, Ospedaletto, 56100 Pisa, Italy; 8 Department of Clinical and Experimental Medicine, University of Pisa, 56100 Pisa, Italy; 9 Chemistry of Life Processes Institute, Northwestern University, Evanston, IL 60611, USA; 10 Mesulam Center for Cognitive Neurology and Alzheimer’s Disease, Feinberg School of Medicine, Northwestern University, Chicago, IL 60611, USA; 11 Les Turner ALS Center, Feinberg School of Medicine, Northwestern University, Chicago, IL 60611, USA; 12 Robert H. Lurie Comprehensive Cancer Center, Northwestern University, Chicago, IL 60611, USA

**Keywords:** alsin, metabolites, serum, plasma, mutation, biomarker

## Abstract

Pathogenic variants in *ALS2* have been detected mostly in juvenile cases of amyotrophic lateral sclerosis (ALS), affecting mainly children and teenagers. Patients with *ALS2* mutations demonstrate early onset cortical involvement in ALS. Currently, there are no effective treatment options. There is an immense need to reveal the underlying causes of the disease and to identify potential biomarkers. To shed light onto the metabolomic events that are perturbed with respect to *ALS2* mutations, we investigated the metabolites present in the serum and plasma of a three-year-old female patient (AO) harboring pathogenic variants in *ALS2*, together with her relatives, healthy male and female controls, as well as another two-year-old patient DH, who had mutations at different locations and domains of *ALS2*. Serum and plasma samples were analyzed with a quantitative metabolomic approach to reveal the identity of metabolites present in serum and plasma. This study not only shed light onto the perturbed cellular pathways, but also began to reveal the presence of a distinct set of key metabolites that are selectively present or absent with respect to *ALS2* mutations, laying the foundation for utilizing metabolites as potential biomarkers for a subset of ALS.

## 1. Introduction

Alsin is a 184 kDa protein of 1657 amino acid residues that is encoded by the *ALS2* gene [1,2]. The presence of several guanine nucleotide exchange factor (GEF) domains, including three main domains, the N-terminal regulator of chromosome condensation 1-like domain (RLD), the central Dbl homology and pleckstrin homology domain (DH/PH), and the C-terminal vacuolar protein sorting 9 domain (VPS9) [3] suggests that *ALS2* acts as a regulator/activator of multiple small GTPases.

More than 100 pathogenic variants in *ALS2* have been reported in patients with motor neuron diseases [3]. The majority of these are nonsense or frameshift mutations, and the resulting mutants are unstable and are degraded via the ubiquitin–proteasome system (UPS) [4] or are nonfunctional due to a lack of the C-terminal Rab5 GEF domain [4]. Recessive mutations in *ALS2* gene lead to a spectrum of motor neuron disorders with a predominantly upper motor neuron (UMN) degeneration signature. These disorders are juvenile amyotrophic lateral sclerosis (JALS), juvenile primary lateral sclerosis (JPLS), and infantile-onset ascending hereditary spastic paraplegia (IAHSP) [1,2,5]. In JPLS, the degeneration is mostly restricted to UMNs [6], whereas in ALS and IAHSP, both upper and lower motor neurons are compromised. As of now, 82 patients with early-onset motor neuron diseases due to variants in *ALS2* have been identified [3,7]. Of these patients, 53 cases (64.6%) were diagnosed with IAHSP. Four cases had JPLS (4.9%), while 25 cases were reported with JALS (30.5%) [7]. The average age of disease onset of IAHSP is 15.5 months. For JPLS, the average age of onset is 24 months, while for JALS, the average age of onset is 4.9 years.

Here, we utilized serum and plasma samples isolated from healthy male and female control subjects, as well as from a three-year-old female with mutations in the *ALS2* gene (*c.4368delG* and *c.4831C>T*), her brother (*c.4368delG*), mother (*c.4831C>T*), and father (*c.4368delG*). In addition, we also had access to the serum and plasma samples of another two-year-old female with a different set of mutations in the *ALS2* gene (*c.529G>C* and *c.3304G>A*) along with her mother (*c.3304G>A*) and father (*c.529G>C*). Interestingly, these novel mutations are located at different regions of the gene and are not reported previously in any of the *ALS2* patients.

Since the alsin protein plays a pivotal role in a wide array of cellular events, such as endocytosis, membrane dynamics, and cytoskeletal organizations, mutations in the *ALS2* gene have significant consequences [8,9,10,11]. To better understand the systemic failures that occur in the presence of *ALS2* mutations and how these are reflected onto the metabolite distribution in serum and plasma, we performed a metabolomic study.

Alterations and perturbations in metabolites are informative about the cellular events that are compromised in patients [12,13,14,15]. Most importantly, metabolites can be developed as biomarkers to detect mechanistic perturbations as well as biomarkers that help distinguish patients based on the underlying causes of the disease [16,17,18].

Our results revealed a distinct set of metabolites that are detected either at high levels or are absent in the serum and/or plasma of patients with *ALS2* mutations. Canonical pathway analyses pointed mainly to mitochondrial defects as one of the major perturbed cellular events. Our study began to reveal the presence of key metabolites that deserve further investigation for their potential to distinguish ALS patients and suggest the involvement of key cellular events in disease pathology.

## 2. Results

### 2.1. Selection of Patients

This report is presented almost as a case study of the patient AO, because metabolomic data were collected from her; her relatives (i.e., mother, father, and brother); healthy male and female subjects; and DH, an age- and sex-matched ALS patient to AO with a different set of mutations in the *ALS2* gene. Since it is not possible to perform proper statistical analyses with only two patients, and because we had information about AO’s relatives, here we present our results within the perspective of patient AO, even though results from DH are also reported.

**Figure 1:**
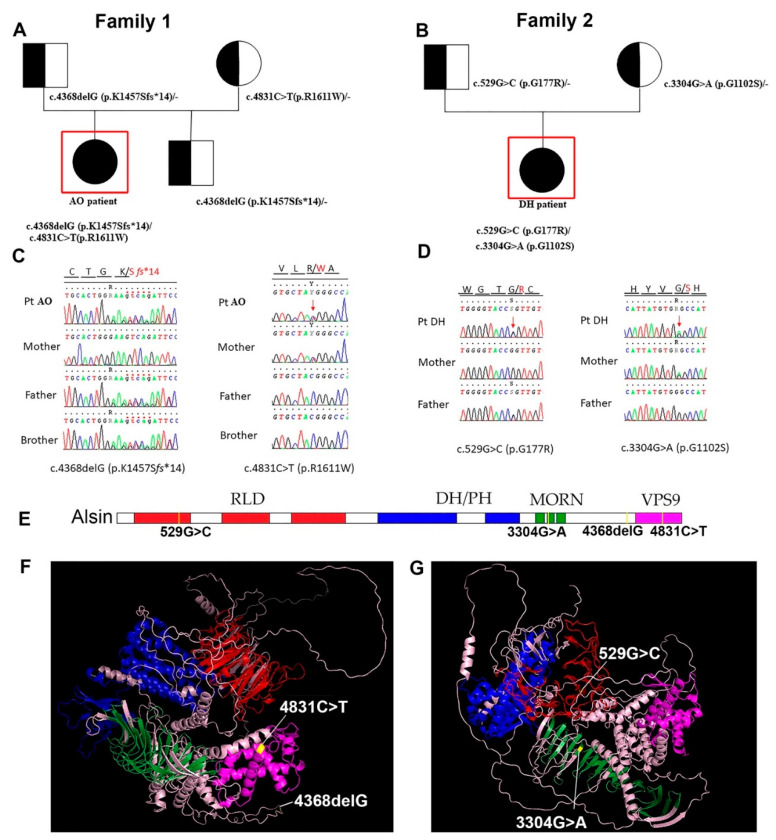
Family pedigrees and the location of mutations in the *ALS2* gene. (**A**,**B**) The family tree of patients AO (**A**) and DH (**B**), who have different mutations in their *ALS2*. (**C**) Gene sequencing results show deletion at *c.4368delG* site and a point mutation at *c.4831C>T* site in Family 1. (**D**) Similarly, gene sequencing data show a point mutation at *c.529G>C* site and a point mutation at *c.3304G>A* site in Family 2. (**E**) Schematic representation of linear structure of *ALS2* gene, highlighting mutations at different sites. (**F**) 3D conformation of alsin protein, showing the location of *c.4368delG* deletion and *c.4831C>T* mutation site. (**G**) 3D conformation of alsin protein showing *c.529G>C* and *c.3304G>A* mutation sites.

Two different and independent families contributed to this study. Family 1 had one daughter and one son, Family 2 had one daughter (Figure 1A,B). Using a NGS HSP gene panel in both probands, we identified two compound heterozygous variants in *ALS2* (NM_020919.4) in AO: the novel *c.4368delG* (p.K1457fs*14), inherited from her father, and *c.4831C>T* (p.R1611W), inherited from her mother, already reported in the HGMD database [19] (Figure 1A–D). Segregation analyses were performed on the family members by Sanger sequencing. According to the American College of Medical Genetics and Genomics (ACMG) guidelines, both variants are likely pathogenic: *c.4831C>T* with evidence of pathogenicity PM2, PM5, PP3 and CADD score of 33.0 and *c.4368delG* with evidence of pathogenicity PVS1, PM2. These variants are ultrarare and have not been previously reported in the gnomAD database.

In the second family, the only daughter DH showed novel biallelic variants in the *ALS2* gene (Figure 1B,D). The *c.529G>C* (p.G177R) arose on the paternal allele, and *c.3304G>A* (p.G1102S) came from the maternal allele. Both variants are likely pathogenic (PM2, PP3) according to ACMG, with CADD scores of 29.60 and 29.90, respectively, and are not previously reported in the gnomAD database. Since DH was age- and sex-matched with AO and they displayed similar disease pathology, we included DH in our analyses to investigate the metabolites that are common and different between AO and DH.

To reduce variability among the control samples, we prepared a pool of male control samples, which included 4 independent healthy male cases, and a pool of female control samples, which included 4 independent healthy female cases. The ages of the males were chosen in close proximity to the paternal parents of AO, while the ages of the females were chosen in close proximity to the maternal parents of AO. Metabolomic analyses were performed on both the serum and plasma samples isolated from AO, DH, and the relatives of AO (i.e., the mother, father, and brother), together with the healthy female and male control samples (*n* = 4 each), as described above.

When analyzing data, emphasis was given to metabolites that were present in both AO and DH but absent in all male and female controls. Likewise, metabolites that were present in all healthy controls, but absent in AO and DH were investigated. The levels of these metabolites in AO’s relatives (brother, father, mother) were also noted. A reading of zero in the control samples meant the lack of any detection in any of the control subjects. The exact same analyses were performed for both serum and plasma samples with at least two experimental replicates.

Since AO and DH have mutations in different regions of the *ALS2* gene and affecting different domains (Figure 1E,F; Supplementary Movie S1), we speculated that this variation may lead to potential differences in the cellular events that are primarily affected, and thus may lead to important information about the disease. Therefore, in a second set of analyses, differences between AO and DH were also investigated.

### 2.2. Metabolites Suggest Potential Defects in Key Canonical Pathways

**Figure 2:**
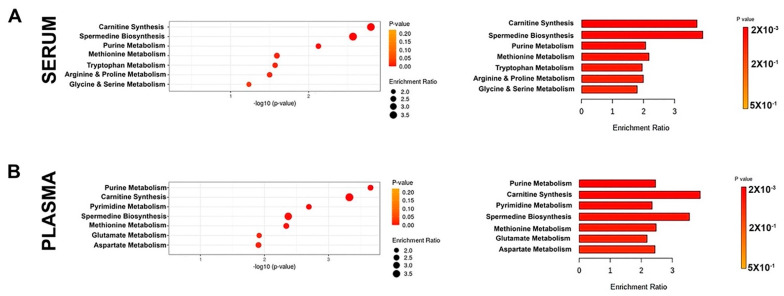
Top canonical pathways that were highlighted based on the metabolites detected in the serum and plasma samples of patients with mutations in their *ALS2* gene. Top 7 canonical pathways are listed based on their significance and enrichment ratio both for metabolites isolated from serum (**A**) and plasma (**B**) samples.

To investigate whether metabolite profiling would inform us about the cellular events that are perturbed in AO and DH, and to gain a better understanding of potential problems that occur in patients, we performed a metabolite enrichment assay with the list of metabolites and their quantitation (Figure 2; Supplemental Table S1). The results suggested the presence of a set of canonical pathways that were determined based on significance (*p* value) and the percent representation of metabolites among all metabolites that are associated with that given canonical pathway (enrichment ratio).

Among all canonical pathways, carnitine synthesis (*p* value = 0.0016), spermidine biosynthesis (*p* value = 0.0027), and purine metabolism (*p* value = 0.0075) were the top three canonical pathways suggested for the metabolites detected in the serum (Figure 2A; Supplementary Table S1). Likewise, purine metabolism (*p* value = 0.00022), carnitine biosynthesis (*p* value = 0.00047), and pyrimidine metabolism (*p* value = 0.002) were the top three canonical pathways identified based on the metabolites present in the plasma (Figure 2B; Supplementary Table S2). Interestingly, carnitine synthesis and purine metabolism were within the top three canonical pathways both for serum and plasma, further suggesting alterations in mechanisms that are important for energy balance and the presence of potential problems regarding the integrity of mitochondria.

### 2.3. Metabolites Present in Serum

The metabolomic analyses revealed the presence of a distinct set of metabolites measured as arbitrary units (au), which were converted to log2 base and quantitatively assessed. Since healthy controls and the parents of AO were analyzed together with AO and DH, the relative values are represented in bar graphs for both serum and plasma. The exact same experimental procedures were performed on the serum and plasma samples, with experimental replicates. Here, we highlight only the most dramatic differences between the healthy controls and the patients. 

**Figure 3:**
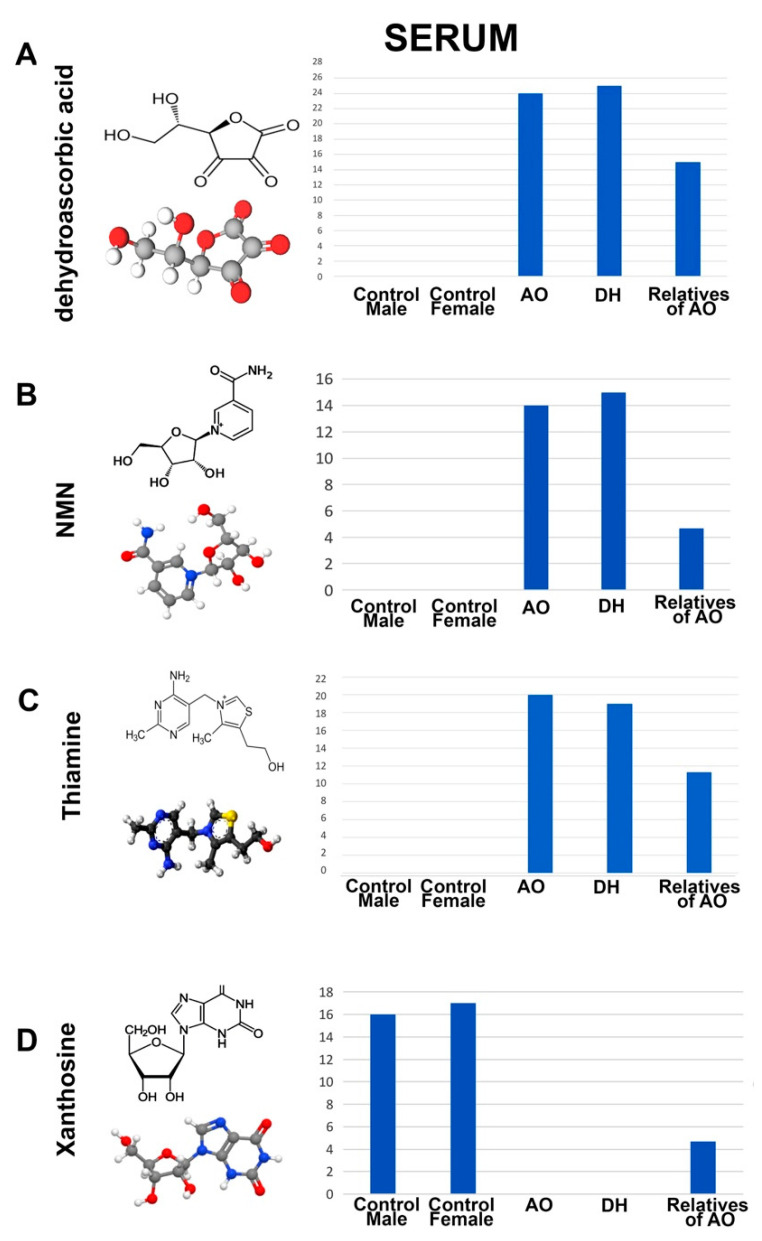
Metabolites that are present or absent from the serum of AO and DH. Molecular structure, ball–stick representation, and bar graph distribution of dehydroascorbic acid (**A**), NMN (**B**), thiamine (**C**), and xanthosine (**D**).

Only a distinct set of metabolites were selectively present in the serum samples of both AO and DH but totally excluded from all healthy controls. Interestingly, the relatives of AO, who were heterozygous for *ALS2* mutations, had lower levels of these metabolites. For example, a set of metabolites were present at high levels in both AO and DH but absent from healthy controls, such as dehydroascorbic acid (AO = 24; DH = 25; AO’s relatives = 15; female controls = 0; male controls = 0), NMN (AO = 14; DH = 15; AO’s relatives = 6; female controls = 0; male controls = 0), and thiamine (AO = 20; DH = 19; AO’s relatives = 11; female controls = 0; male controls = 0). On the contrary, xanthosine was present at high levels in the both the male and female control samples but was completely missing in the serum samples isolated from AO and DH (AO = 0; DH = 0; AO’s relatives = 10; female controls = 17; male controls = 16 (Figure 3)).

In addition to the metabolites that were selectively present in the serum samples isolated from AO and DH, there were also a distinct set of metabolites that were differentially present either in AO or in DH. Since the *ALS2* mutations were at different locations in AO and DH—more specifically, in the VP59 region in AO and in the MORN and RLD regions in DL (Figure 1)—we anticipated that these mutations located at different regions and motifs potentially affected different functions of the same alsin protein. Thus, we investigated the presence of metabolites that were differentially present between AO and DH.

**Figure 4:**
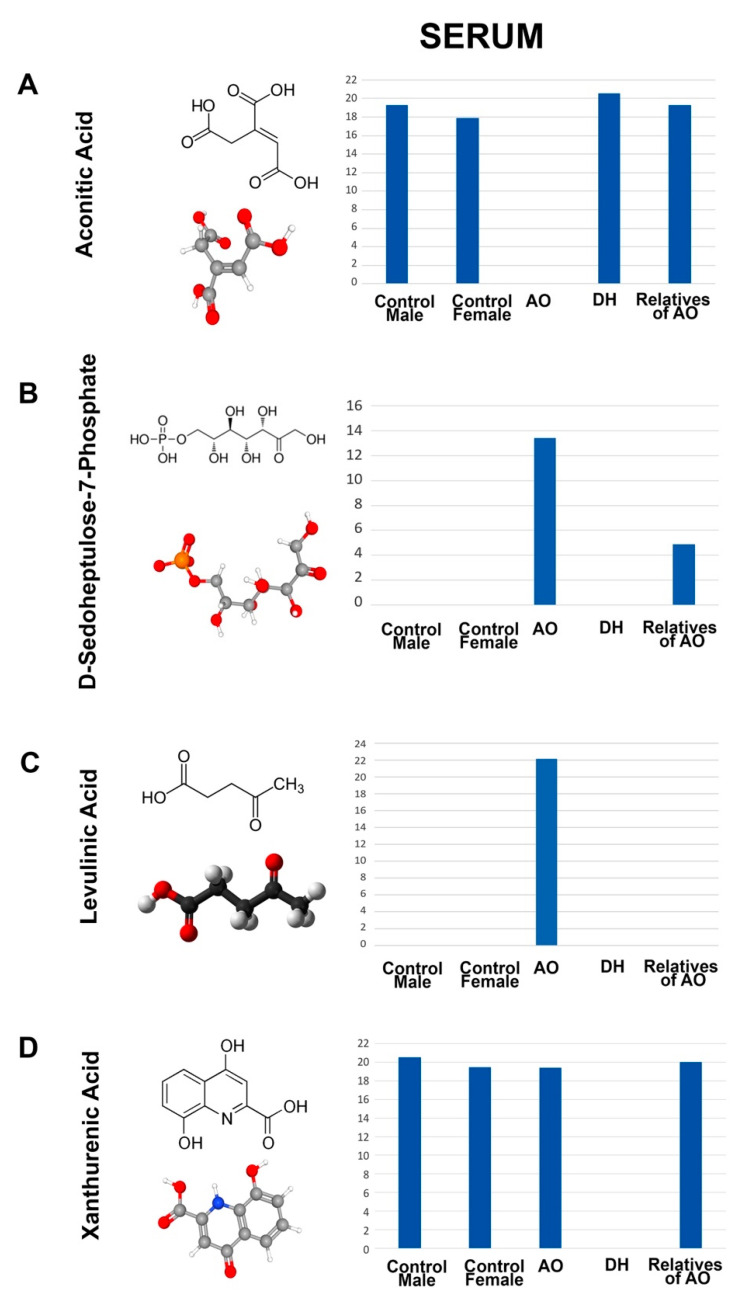
Metabolites that show differential presence in the serum of AO and DH. Molecular structure, ball–stick representation, and bar graph distribution of aconitic acid (**A**), D-sedothepulose-7-phosphate (**B**), levulinic acid (**C**), and xanthurenic acid (**D**).

There was no metabolite that was present only in DH. However, aconitic acid was present in DH, absent in AO, and also present in the healthy controls and the parents of AO (AO = 0; DH = 21; AO’s relatives = 19; female controls = 18; male controls = 19). Most interestingly, there were two metabolites that were present only in the serum of AO: D-sedothepulose-7-phosphate (AO = 13; DH = 0; AO’s relatives = 6; female controls = 0; male controls = 0) and levulinic acid (AO = 22; DH = 0; AO’s relatives = 0; female controls = 0; male controls = 0). On the contrary, xanthurenic acid was absent only in DH (AO = 19; DH = 0; AO’s relatives = 20; female controls = 19; male controls = 21). It is interesting that even though AO and DH have mutations in the same gene, there are differences in the profile of their serum metabolites (Figure 4).

### 2.4. Metabolites Present in Plasma

**Figure 5:**
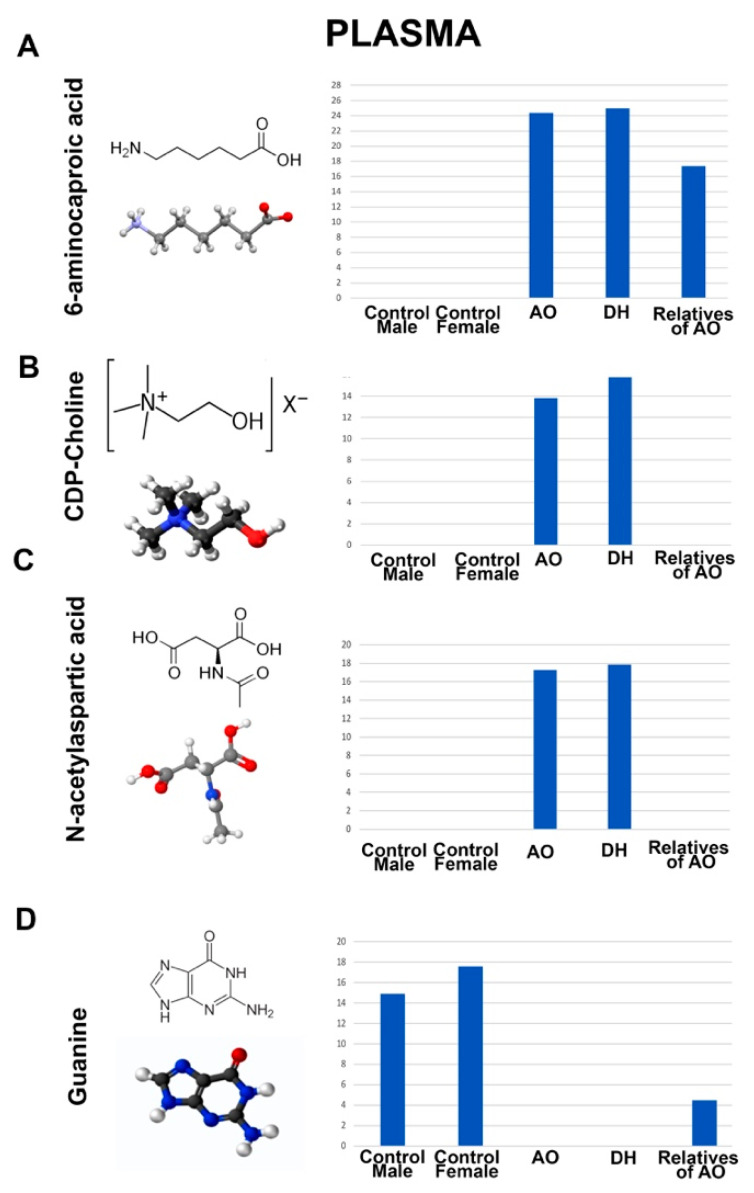
Metabolites that are present or absent from the plasma of AO and DH. Molecular structure, ball–stick representation, and bar graph distribution of 6-aminocaproic acid (**A**), choline (**B**), N-acetylaspartic acid (**C**), and guanine (**D**).

To better understand the metabolite distribution in blood, we investigated the plasma with the same parameters and experimental approach and in parallel to the serum samples isolated from AO, DH, the healthy male and female controls, and the relatives of AO. Interestingly, the metabolites detected in the plasma were mostly different from those detected in the serum (Figure 5). For example, 6-aminocaproic acid was present in 

**Figure 6:**
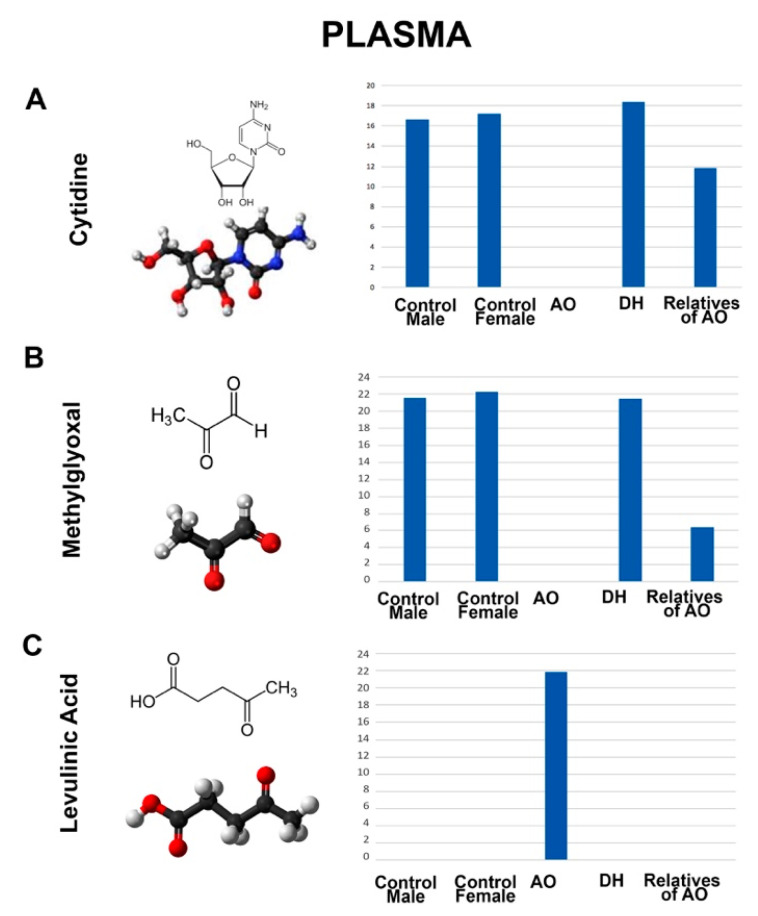
Metabolites that show differential presence in the plasma of AO or DH. Molecular structure, ball–stick representation, and bar graph distribution of cystidine (**A**), levulinic acid (**B**), and methylglyoxal (**C**).

We also detected metabolites that showed differential presence between the plasma isolated from the blood samples of AO and DH (Figure 6). For example, cytidine (AO = 0; DH = 18; AO’s relatives = 16; female controls = 17; male controls = 17) and methylglyoxal (AO = 0; DH = 21; AO’s relatives = 6; female controls = 22; male controls = 22) were not detected in the plasma of AO but were present in the plasma of DH and relatives of AO. Interestingly, levulinic acid, which was also detected in the serum of AO, was also present in the plasma (and only in the plasma) of AO, and was totally absent in the plasma of DH or the relatives of AO (AO = 22; DH = 0; AO’s relatives = 0; female controls = 0; male controls = 0).

These results revealed the dynamic range of metabolites that are present in the serum and plasma samples isolated from two different juvenile ALS patients with distinct *ALS2* mutations, and further suggested that not only the mutated gene, but also the location of the mutation is informative.

## 3. Discussion

ALS is one of the most complex neurodegenerative diseases, with many different underlying causes [20]. The genetics of ALS reveal the heterogenous, multifactorial nature of motor neuron disease, which affects both the cortical and the spinal components of motor neuron circuitry [21,22]. Among all mutations detected in ALS patients, *ALS2* mutations are of significance, mostly because they result in early-onset ALS, observed in young adults and even in children [2,5]. 

Alsin has numerous different functions and interacts with itself through the C-terminal region, forming a homophilic oligomer [23]. This homo-oligomerization is the key to Rab5 GEF activity and alsin-mediated endosome fusion [10,23]. Specifically, alsin acts as a GEF for the small GTPase Rab5 via the C-terminal MORN/VPS9 domain, which modulates endosome fusion and trafficking by activating Rab5 [24]. The activation of Rab5 is essential for protein trafficking through the early stages of the endocytic pathway [25].

The loss of alsin function results in the delayed fusion of epidermal growth factor (EGF)-positive endosomes in mouse embryonic fibroblasts (MEFs) [8], thus supporting the idea that endogenous alsin is involved in Rab5-dependent endosome fusion. The alsin protein is proposed to have several guanine nucleotide exchange factor (GEF) domains and be able to activate Rho, Rac1, and Rab5 GTPases, which play important roles in endocytosis, cytoskeleton maintenance, protein transport, cell signaling, neurite outgrowth, and membrane trafficking [1,2,26,27]. Additionally, the overexpression of alsin results in abnormal endosomal phenotypes, the impairment of mitochondria trafficking, and the fragmentation of the Golgi apparatus [28].

Alsin is ubiquitously expressed and is vital especially for motor neurons [8,9,10]. Thus, the dysregulation of alsin function would impact a wide array of proteins and other metabolites. Here, we report a comprehensive analysis of the metabolome of both serum and plasma samples with respect to mutations in the alsin gene.

Metabolomic studies are gaining momentum for revealing the extent of systemic perturbations that occur in patients. Many different metabolites are detected in the serum and plasma of blood and other biological specimens, suggesting and revealing problems with or limitations to cellular function, organ failure, and the potential causes of diseases. The investigation of metabolites in ALS is an emerging topic. Thus, studying the metabolite profile of both serum and plasma is especially important for juvenile ALS patients with *ALS2* mutations. 

Since ALS is very heterogeneous, reaching statistical significance requires the inclusion of very many different patients in studies. This is a major limiting factor, because ALS is a rare disease and having access to a large population of patients at a given time is not always feasible. Therefore, well-defined case studies are exceptionally important. Even though the sample size is very small, case studies inform on the biological events that occur specifically in that patient, sometimes revealing key proteins, molecules, and biological events that need further analyses and investigation in a larger cohort of patients. These well-defined case studies lay the foundation for larger and hypothesis-driven studies to investigate the validity of results in a larger patient population.

Our study began to reveal the presence of key metabolites that showed striking differences between patients (i.e., AO, DH) and controls (ie. relatives of AO, and healthy male and female controls). For example, we found that xanthosine and guanine were depleted in both AO and DH, and that N-acetylaspartic acid and choline were only present in AO and DH. It is important to further investigate whether these metabolites may serve to distinguish alsin patients among other ALS patients and whether these metabolites help inform about cellular dysfunctions related to specific mutations.

Proteins such as alsin have numerous different functions and many functional domains. Mutations in one particular domain would impair the protein activity dependent on that domain, whereas functions manifested by other domains would continue undisturbed. Therefore, even though two different patients have mutations in one common gene, depending on where the mutations are, they may have different aspects of disease manifestations. Therefore, our comparative metabolite profiling between AO and DH is rather interesting and revealing.

We began to identify metabolites that were differentially present between AO and DH, who have mutations in the same gene but in different locations and domains. This is of great importance for understanding the potential limitations that are caused by distinct mutations affecting different domains. We found that, different from other metabolites, levunilic acid was present at high levels both in the serum and plasma of AO, while it remained undetected in the serum and plasma samples isolated from DH, all female and male controls, and the relatives of AO.

Levunilic acid was very specific to AO. Levulinic acid is a straight-chain saturated fatty acid and an oxopentanoic acid, which is important in that it has both keto and acid groups and the potential to produce numerous additional fuel additives such as γ-valerolactone (GVL), 2-methyltetrahydrofuran (MTHF), and ethyl levulinate [29,30,31]. It is important for ATP production, especially when the electron transport chain becomes dysfunctional and the cell requires other alternatives to produce the energy required. This suggests potential problems with energy production and mitochondrial health, and that the body is trying to cope with the problem by increasing the levels of levunilic acid.

To further understand the biology and pathology behind the metabolomic alterations, we need to highlight the importance of the metabolites that were differentially present or absent in the serum or plasma of AO and DH. Table 1 summarizes the most important functions of the metabolites detected and highlighted in this study.

Even though there were differences between the location of the mutations in *ALS2*, some metabolites were present in the serum and plasma samples of both AO and DH but absent in the healthy controls, suggesting the presence of some shared aspects of the disease. For example, 5’-Methylthioadenosine, dehyroascorbic acid, nicotinamide mononucleotide, and thiamine were present in the serum of AO and DH, whereas 6-aminocaproicacid, choline, and N-acetylaspartic acid were present in the plasma of AO and DH but were excluded from the healthy controls. On the contrary, guanine was missing from the plasma of AO and DH, and xanthosine was not detected in the serum of AO and DH.

The differences between the metabolites began to reveal perturbations that occur in juvenile ALS patients with *ALS2* mutations. In particular, the enriched metabolite analyses highlighted the involvement of carnitine synthesis. Carnitine is critically important for various cellular functions. For example, it shuttles activated long-chain fatty acids into the mitochondria so that β-oxidation can take place. This path is taken when there are problems with the inner mitochondrial membrane and the electron transport chain cannot be utilized to generate ATP. Mitochondria switch to the breaking down of lipids and sugar moieties as an alternative path to generate ATP. We found that, in addition to numerous metabolites that are related to alternative paths for generating energy, the carnitine synthesis was detected by the metabolites present in both the serum and plasma.

These results ultimately suggest a perturbed metabolite balance with a special emphasis on mitochondrial problems and limitations to energy production. The changes in the metabolite profiles of the serum and plasma of AO and DH suggest the presence of prominent metabolomic perturbations even at this very young age and that mitochondrial dynamics are affected in the disease. 

Since mutations in the *ALS2* gene represent one of the most significant defects that lead to juvenile cases of ALS, our results suggest the importance of maintaining the health and function of mitochondria. It is possible that maintaining metabolite balance, improving mitochondrial health and function, as well as establishing metabolomic homeostasis at a cellular and systems level would offer novel therapeutic interventions, especially for juvenile and severe cases of ALS.

## 4. Materials and Methods

### 4.1. Patient Information

**AO and Family 1:** AO is a 3-year-old female. She was born after an uneventful pregnancy to healthy and unrelated parents. Her two-year-old brother was also healthy. Birth was unremarkable. The patient sat up unsupported at the age of 6 months, with subsequent crawling at 8 months and independent walking at 12 months. At 15 months, she manifested unsteady gait and repeated falls; leg stiffness and walking on tiptoes were observed by parents from 18 months of age. Patient’s complete blood count and biochemical parameters were normal. At the age of 22 months, brain and spinal cord magnetic resonance imaging showed no abnormalities. Electroencephalogram was normal. The cognitive subscale of the Bayley Scale of Infant Development III, performed at 22 months of age, showed a normal cognitive development. At two years, neurological examination revealed an abnormal gait with tiptoe walking due to spastic hypertonia of the lower limbs; enhanced deep-tendon reflexes, bilateral ankle clonus, and Babinski sign were present bilaterally. She could control her head and maintain the sitting position and she also showed good coordination and hand manipulation. She was able to achieve a full range of eye movements with no nystagmus. She had mild sialorrhea due to the facial weakness; dysarthria was also observed. The parents did not report any sphincter dysfunction in the patient’s bladder and bowel functions. SNP–CGH array analysis excluded the presence of genomic rearrangements and loss of heterozygosity regions.

**DH and Family 2:** DH is a 2-year-old female. She is the first child of healthy non-consanguineous Italian parents. She was born at term after an uneventful pregnancy by spontaneous vaginal delivery. The newborn period appears to have been uneventful. The patient sat up unsupported at the age of 7 months, started to crawl at 9 months of age, and walked independently at 18 months. Parents described a wide-based gait with frequent falls. At 20 months, following a febrile episode, the patient began dragging her right foot, which slowly progressed to bilateral toe walking and a wide-based stance. Her metabolic testing and motor/sensory nerve conduction velocity studies were normal. Neuroimaging studies showed lipoma of the filum terminale, but no brain MRI abnormalities. Clinical examination at 2 years of age revealed an abnormal gait with knees flexed due to spastic hypertonia of the lower limbs. Proximal weakness was observed in the lower limbs (quadriceps and gluteal muscles). Other manifestations of pyramidal syndrome, hyperreflexia, clonus, and Babinski sign were also present but only in the lower limbs. There were no signs of cerebellar dysfunction. Oculomotor movements presented a normal pattern. The assessment of cognitive abilities was performed using Wechsler Preschool and Primary Scale of Intelligence, third edition (WPPSI III), which showed an Intelligence Quotient (IQ) of 117.

**Control Cases:** 4 independent healthy males were included in the study (i.e., CB—age 48; FT—age 39; FS—age 35; IS—age 50). Serum and plasma were isolated from the blood samples collected from each control male, and all control serum samples were pooled and analyzed together as one control case.

Four independent females were included in the study (i.e., FY—age 63; HT—age 26; NY—age 42; NA—age 45). Serum and plasma were isolated from the blood samples collected from each control female, and all control serum samples were pooled and analyzed together as one control case.

### 4.2. Genetic Analyses

Genomic blood DNA from the two patients was purified by standard methods and analyzed using Spastisure 4.0, a customized TRP able to investigate the coding regions of 130 genes linked to HSP, spastic ataxias, or related motor diseases. Candidate pathogenic variants in *ALS2* (NM_020919.4) were confirmed by Sanger sequencing and shown to segregate in the families.

### 4.3. Plasma and Serum Sample Collection

3 mL of whole blood were drawn from patients and collected into EDTA-treated or -free test tubes to obtain plasma or serum, respectively. Both test tubes were centrifuged at 150× *g* for 15 min at room temperature. At the end of the centrifugation step, three different layers were visible in the EDTA-treated tubes: plasma in the upper yellowish layer, the intermediate buffy coat with leucocytes, and the lower layer containing red blood cells. Plasma was aspirated, transferred into a new Eppendorf, and centrifuged at 9600× *g* for ten minutes at room temperature to separate plasma from debris. At the end, 1 mL of plasma was saved and stored at −80 °C. Two layers were visible in the EDTA-free test tubes: the upper yellowish liquid (serum) and the lower coat. 1 mL of serum was aspirated, discarding the lower coat, and transferred into a new Eppendorf, stored at −80 °C.

### 4.4. Metabolomics

#### 4.4.1. Metabolite Extraction

One hundred microliters of serum/plasma was mixed with 900 µL chilled 80% chromatography-grade methanol, and vigorously vortexed three times. Samples were incubated in the -80 freezer overnight. After the incubation, samples were vortexed three times (30 sec each vortex) and centrifuged at 20,000× *g* for 30 min at 4 °C. The supernatant containing metabolites was separated and used for the following step.

#### 4.4.2. Sample Reconstitution after Extraction

Extraction solution was dried using SpeedVac (Thermo Savant, Waltham, MA, USA). Fifty-percent acetonitrile was added to the tube for reconstitution, followed by vortexing for 30 sec. Sample solution was then centrifuged for 15 min at 20,000× *g*, 4 °C. Supernatant was collected for LCMS analysis.

#### 4.4.3. Full-Scan Hydrophilic Metabolite Profiling

Samples were analyzed by High-Performance Liquid Chromatography and High-Resolution Mass Spectrometry and Tandem Mass Spectrometry (HPLC-MS/MS). Specifically, the system consisted of a Thermo Q-Exactive in line with an electrospray source and an Ultimate3000 (Thermo Fisher Scientific, Waltham, MA, USA) series HPLC consisting of a binary pump, degasser, and auto-sampler outfitted with an Xbridge Amide column (Waters Inc., Milford, MA, USA; dimensions of 4.6 mm × 100 mm, and a 3.5 µm particle size). The mobile phase A contained 95% (vol/vol) water, 5% (vol/vol) acetonitrile, 20 mM ammonium hydroxide, 20 mM ammonium acetate, and pH = 9.0; B was 100% acetonitrile. The gradient was as following: 0 min, 15% A; 2.5 min, 30% A; 7 min, 43% A; 16 min, 62% A; 16.1–18 min, 75% A; 18–25 min, 15% A with a flow rate of 400 μL/min. The capillary of the ESI source was set to 275 °C, with sheath gas at 45 arbitrary units, auxiliary gas at 5 arbitrary units, and the spray voltage at 4.0 kV. In positive/negative polarity switching mode, an *m*/*z* scan range from 70 to 850 was chosen and MS1 data were collected at a resolution of 70,000. The automatic gain control (AGC) target was set at 1 × 10^6^ and the maximum injection time was 200 ms. The top 5 precursor ions were subsequently fragmented, in a data-dependent manner, using the higher energy collisional dissociation (HCD) cell set to 30% normalized collision energy in MS2 at a resolution power of 17,500. Data acquisition and analysis were carried out by Xcalibur v4.1 software and Tracefinder software version v4.1, respectively (both from Thermo Fisher Scientific, Waltham, MA, USA).

#### 4.4.4. Metabolite Enrichment Analysis

MetaboAnalyst version 5.0, a web-based platform for enrichment analysis of metabolomic data was used for analysis of processed High-Resolution Mass Spectrometry and Tandem Mass Spectrometry (HPLC/MS) data [57].

## 5. Conclusions

Here, we reported a distinct set of metabolites that displayed striking differences between healthy controls and two juvenile Italian ALS patients with novel *ALS2* pathogenic variants. These mutations have not been previously reported in any juvenile ALS patients with *ALS2* mutations. Since the location of the mutations were at different sites and in different domains, we also detected differences between AO and DH. Metabolites that were present in the serum and plasma differed greatly, with the exception of levunilic acid, which was present only in AO in both serum and plasma samples. This metabolite may offer a signature to detect patients with *ALS2* mutations in the VP59 domain. Overall, the metabolomic analyses highlighted perturbations in the energy metabolism and problems with mitochondria, the electron transport chain, and the generation of ATP. The cells and neurons in our body appear to solve this problem via activating different mechanisms to generate the required ATP and enhance the β-oxidation of fatty acids.

These results reveal the importance of understanding metabolomic dysfunction in ALS patients. The information obtained directly from the biosamples isolated from the patients inform us about systemic perturbations and suggest the presence of a unique signature of metabolites that may help identify the underlying causes of the disease.

It is also important to note that the location of the mutation is almost as important as the gene that is mutated. Since mutations affect different domains within the same gene, it is possible that different cellular functions are perturbed or affected in patients. As the medical field is moving towards personalized medicine approaches, it is becoming more important to understand the pathologies at a personal level. Therefore, the differences between AO and DH are rather interesting, as they begin to shine a light on the metabolomic differences that occur due to the differences at the site of the mutation. Our study paves the way for more detailed studies to be performed and sets the stage for metabolomic analyses, especially for juvenile ALS patients with *ALS2* mutations.

## Figures and Tables

**Table 1:** Key metabolites and their functions.

Metabolite	Function	References
Levulinic acid	Important for ATP production when ETC becomes dysfunctional	[29,30,31]
Aconitic acid	Intermediate in the citric acid cycle, energy production	[32,33]
Sedoheptulose 7-phosphate	Produces ADP and sedoheptulose 7-phosphate, intermediate in the respiratory pathways, generation of energy, reduction of proinflammatory markers	[34,35,36]
Xanthurenic acid	Potent vesicular glutamate transporter (VGLUT) inhibitor, involved in neuronal function and firing potentials	[37,38,39]
Cytidine	Controls neuronal-glial glutamate cycling, helping decrease glutamate/glutamine ratio	[40]
Methylglyoxal	Antiviral, anti-inflammatory, and antioxidant	[41,42]
Dehydroascorbic acid	Protective against viruses, involved in metabolic disorder tyrosinemia type I	[43,44,45]
Nicotinamide mononucleotide	Key intermediate metabolite in the nicotinate and nicotinamide metabolism pathway, energy production	[46,47]
Thiamine	Acts as coenzyme, involved in 2-methyl-3-hydroxybutyryl-coadehydrogenase deficiency pathway	[48]
Xanthosine	Intermediate in purine metabolism, involved in purine nucleoside phosphorylase deficiency	[49]
6-aminocaproic acid	Coagulation, shaping and modulation of plasma proteins	[50,51]
CDP-choline	Component of cell membranes, generation of acetylcholine, production of homocysteine	[52,53,54]
*N*-Acetylaspartic acid	Fluid balance in the brain, energy production	[55,56]

## Data Availability

Data available on request due to privacy restrictions. The data presented in this study are available on request from the corresponding author. The data are not publicly available because they were obtained from patients with consent to be used for this study.

## Supplementary Materials

The following are available online at https://www.mdpi.com/article/10.3390/metabo12020174/s1, Supplementary Movie S1: Three-dimensional structure of the alsin protein with functionally important domains highlighted in color and site of mutations marked with yellow, Supplementary Table S1: Comprehensive list of all metabolites detected in the serum of control and patient samples, Supplementary Table S2: Comprehensive list of all metabolites detected in the plasma of control and patient samples.
