# Supplementary material for: 2-Year-Old and 3-Year-Old Italian ALS Patients with Novel ALS2 Mutations: Identification of Key Metabolites in Their Serum and Plasma
Source: Metabolites. 2022 Feb 12;12(2):174. doi: 10.3390/metabo12020174 (PMC8878019; doi:10.3390/metabo12020174)
Supplement: Supplementary file 1 [file metabolites-12-00174-s001.zip › SuppTable S1-Serum.pdf]

	KEGG ID	Control-males	Control-females	AO-Serum	DH-Serum	Relatives of AO
(S)-4-hydroxymandelonitrile +	C03742	15	16	17	18	17
2-HG	C02630	20	21	18	21	20
2-hydroxybutanoic acid	C05984	0	0	0	0	0
2-keto-isovaleric acid	C00141	28	25	29	30	29
2-oxobutanoic acid	C00109	0	0	0	0	0
3-hydroxy-anthranilic acid	C00632	20	20	20	20	20
3-hydroxykynurenine	C02794	14	15	18	19	17
3-methoxytyramine	C05587	0	0	0	0	0
3-phospho-serine	C01005	13	13	14	13	14
3-ureidopropionic acid	C02642	18	20	18	19	17
3,4-dihydroxymandelic acid	C05580	17	16	15	0	5
3,4-dihydroxyphenylpropanoat	C10447	19	18	16	19	17
4-trimethylammoniobutanoate	C01181	25	24	23	24	23
5-methyl-THF	C00440	0	0	0	0	0
5-methylcytosine	C02376	21	22	20	21	21
5'-methylthioadenosine	C00170	0	0	18	18	19
6-aminocaproic acid	C02378	24	0	0	25	25
6-O-methylguanine	n/a	0	0	13	0	0
6-phosphogluconic acid	C00345	15	14	15	15	16
a-KG	C00026	23	20	22	22	24
acetylcholine+	C01996	22	23	21	22	22
aconitic acid	C00417-C02341	19	18	0	21	19
adenine	C00147	22	22	22	22	22
adenosine	C00212	17	14	17	19	19
adenylosuccinate	C03794	0	0	0	0	0
AICA ribonucleotide	C04677	0	0	0	0	0
alanine	C00041-C00133	30	30	28	28	28
allantoin	C01551	25	25	22	22	24
arginine	C02385	32	33	31	32	31
asparagine	C16438	24	23	25	25	24
aspartic acid	C16433	26	27	22	22	23
b-alanine	C00099	0	0	0	0	0
betaine	C00719	31	31	30	31	31
carnitine+	C00487	30	31	30	30	30
CDP	C00112	0	0	0	0	0
CDP-choline	C00307	0	0	0	0	6
choline+	C00114	32	33	29	30	30
citraconic acid	C02226	24	21	23	24	24
citrulline	C00327	24	24	24	24	23
CMP	C00055	0	0	0	0	5
creatine	C00300	27	28	29	29	28
creatinine	C00791	31	31	29	30	28
CTP	C00063	0	0	0	0	0
cystathionine	C00542	20	20	19	20	22
cysteine	C00736-C00097	0	18	19	20	18
cysteine-S-sulfate	C05824	21	22	23	24	22
cystine	C01420	26	26	27	28	27
cytidine	C00475	18	17	16	20	18
cytosine	C00380	20	21	19	20	20
D-arabinono-1,4-lactone	C00652	22	22	23	24	22
D-erythrose-4-phosphate	C00279	0	0	0	0	0
D-sedoheptulose-7-phosphate	C05382	0	0	13	0	6
dADP	C00206	0	0	0	0	0
dAMP	C00360	0	0	0	0	0
dATP	C00131	0	0	0	0	0
dCMP	C00239	0	0	0	0	0
dCTP	C00458	0	0	0	0	0
dehydroascorbic acid	C05422	0	0	24	25	15
deoxyguanosine	C00330	0	0	0	0	0
deoxyinosine	C05512	15	15	0	0	5
deoxyuridine	C00526	18	0	18	19	17
dihydroorotate	C00337	18	18	21	20	20
dimethylglycine	C01026	0	0	0	0	0
DL-DOPA	C00355	18	18	17	17	17
dopamine	C03758	0	0	0	0	0
dTMP	C00364	0	0	0	0	0
dTTP	C00459	0	0	0	0	0
dUMP	C00365	0	0	0	0	0
epinephrine	C00788	20	20	20	20	19
FAD	C00016	0	0	0	0	0
FMN	C00061	0	0	0	0	0
folic acid	C00504	0	0	0	0	0
fumaric acid	C00122	20	20	20	20	21
GABA	C00334	20	19	20	18	20
GDP	C00035	0	0	0	0	0
GDP-L-fucose	C00325	0	0	0	0	0
glucono-lactone	C00198	0	0	0	0	0
glutamine	C00303	27	27	29	29	27
glutaminic acid	C00302	29	29	25	24	25
glutathione	C00051	0	0	0	0	10
glutathione disulfide	C00127	18	18	19	19	19
glyceric acid	C00258	29	29	27	28	28
glycine	C00037	27	27	26	26	26
glycylglycine	C02037	17	0	20	0	12
GMP	C00144	0	0	0	13	9
GTP	C00044	0	0	0	0	0
guanidine	C17349	18	17	17	18	17
guanidinoacetate	C00581	23	23	22	20	23
guanine	C00242	18	0	18	19	19
guanosine	C00387	15	0	22	21	21
histamine	C00388	0	0	0	0	0
histidine	C00768	28	28	27	27	27
homocysteine	C05330	24	24	22	25	23
hydroxyphenyllactic acid	C03672	24	24	23	23	24
hypoxanthine	C00262	30	28	27	27	25
IDP	C00104	0	14	15	14	11
imidazole-4-acetate	C02835	23	24	22	23	23
IMP	C00130	13	17	14	14	15
inosine	C00294	19	0	24	23	22
kynurenic acid	C01717	20	20	19	19	20
kynurenine	C01718	25	26	25	26	26
L-arginino-succinate	C03406	19	20	19	19	19
L-cysteic acid	C00506	0	0	0	0	0
L-NMMA	C03884	26	26	25	26	26
L-tyrosine methyl ester	C03404	16	14	15	14	10
lactic acid	C01432	0	0	0	0	0
levulinic acid	n/a	0	0	22	0	0
lysine	C00047/C00739	32	32	30	31	31
maleic acid	C01384	0	17	16	0	0
malic acid	C00711	25	25	24	24	25
methionine	C00073	29	29	28	29	28
methylglyoxal	C00546	22	21	19	0	7
methylmalonic acid	C02170	26	26	25	26	25
mevalonic acid	C00418	23	23	22	22	24
N-acetyl-DL-alanine	n/a	26	26	25	26	26
N-acetyl-L-glutamic acid	C00624	0	0	0	0	11
N-acetylaspartic acid	C01042	0	17	0	20	6
N-acetylaspartylglutamic acid	C12270	17	18	19	19	17
N-carbamoyl-L-aspartate	C00438	0	0	0	0	0
N-methyltryptamine	C06213	13	17	16	15	17
NAD+	C00003	0	0	0	0	6
NADPH	C00005	0	13	0	0	0
nicotinamide	C00153	24	23	23	24	23
NMN	C00455	0	0	14	15	6
octopamine	C04227	20	20	19	20	19
ornithine	C01602	29	29	27	27	28
orotic acid	C00295	22	22	20	21	21
orotidylic acid	C01103	0	0	0	0	0
oxalacetic acid	C00036	0	0	0	0	0
pantothenic acid	C00864	23	23	23	25	23
phenethylamine	C05332	0	0	0	0	0
phenylalanine	C02057	32	32	30	30	31
phosphocreatine	C02305	14	0	19	20	18
phosphoenolpyruvate	C00074	0	0	0	0	0
phosphorylcholine+	C00588	21	21	21	21	22
picolinic acid	C10164	21	22	21	21	21
proline	C16435	32	33	32	32	31
PRPP	C00119	0	0	0	0	0
pseudouridine	C02067	24	24	24	25	23
putrescine	C00134	17	0	17	18	16
pyridoxine	C00314	21	20	18	19	19
pyruvic acid	C00022	0	0	0	0	0
quinolinic acid	C03722	17	17	18	18	18
S-adenosyl-L-homocysteine	C00021	0	0	0	0	0
S-adenosyl-L-methionine+	C00019	13	0	18	19	20
serine	C00716	24	24	23	23	23
serotonin	C00780	0	0	17	0	0
spermidine	C00315	26	26	27	27	26
spermine	C00750	23	23	25	24	24
succinic acid	C00042	24	24	22	23	24
taurine	C00245	30	30	29	29	29
thiamine+	C00378	0	0	20	19	11
thymidine	C00214	18	16	16	17	16
thymine	C00178	21	22	21	20	20
tryptamine	C00398	0	17	18	17	18
tryptophan	C00806	30	30	30	30	30
tyramine	C00483	20	20	20	18	19
tyrosine	C01536	30	30	30	30	29
UDP	C00015	0	0	0	0	0
UDP-D-glucose	C00029	0	0	0	0	6
UMP	C00105	0	0	0	0	5
uracil	C00106	23	23	21	21	23
uric acid	C00366	30	30	30	31	30
uridine	C00299	25	26	24	25	24
UTP	C00075	0	0	0	0	0
valine	C16436	31	31	30	31	30
xanthine	C00385	26	26	25	25	25
xanthosine	C01762	16	17	0	0	10
xanthurenic acid	C02470	21	19	19	0	20
XMP	C00655	0	0	0	0	0
y-glutamylcysteine	C00669	0	0	0	0	0
