# Supplementary material for: 2-Year-Old and 3-Year-Old Italian ALS Patients with Novel ALS2 Mutations: Identification of Key Metabolites in Their Serum and Plasma
Source: Metabolites. 2022 Feb 12;12(2):174. doi: 10.3390/metabo12020174 (PMC8878019; doi:10.3390/metabo12020174)
Supplement: Supplementary file 1 [file metabolites-12-00174-s001.zip › SuppTable S2-Plasma.pdf]

	KEGG ID	Control-Males	Control-Femeales	AO-Plasma	DH-Plasma	AO's Relatives
**(S)-4-hydroxymandelonitrile +**	**C03742**	17	15	16	17	16
**2-HG**	**C02630**	20	19	18	19	19
**2-keto-isovaleric acid**	**C00141**	29	28	29	30	29
**3-hydroxy-anthranilic acid**	**C00632**	19	19	20	21	21
**3-hydroxykynurenine**	**C02794**	16	14	18	19	18
**3-phospho-serine**	**C01005**	20	19	20	19	19
**3-ureidopropionic acid**	**C02642**	18	19	17	19	17
**3,4-dihydroxymandelic acid**	**C05580**	15	17	15	0	5
**3,4-dihydroxyphenylpropanoate**	**C10447**	19	16	15	13	17
**4-trimethylammoniobutanoate+**	**C01181**	24	24	22	23	22
**5-methylcytosine**	**C02376**	21	21	20	21	20
**5'-methylthioadenosine**	**C00170**	14	0	18	18	18
**6-aminocaproic acid**	**C02378**	0	0	24	25	17
**6-phosphogluconic acid**	**C00345**	15	14	18	20	19
**a-KG**	**C00026**	24	22	21	23	22
**acetylcholine+**	**C01996**	23	22	21	22	21
**aconitic acid**	**C00417-C02341**	15	18	20	21	21
**adenine**	**C00147**	22	21	22	22	22
**adenosine**	**C00212**	14	14	16	20	14
**adenylosuccinate**	**C03794**	15	0	0	0	0
**alanine**	**C00041-C00133**	29	30	27	27	27
**allantoin**	**C01551**	26	25	22	23	23
**arginine**	**C02385**	32	32	30	31	31
**asparagine**	**C16438**	23	23	25	25	26
**aspartic acid**	**C16433**	25	26	25	24	26
**betaine**	**C00719**	30	30	30	31	31
**carnitine+**	**C00487**	30	31	28	29	28
**CDP**	**C00112**	16	14	17	15	11
**CDP-choline**	**C00307**	0	0	14	16	0
**choline+**	**C00114**	32	33	29	30	29
**citraconic acid**	**C02226**	22	22	22	22	24
**citrulline**	**C00327**	24	24	24	24	24
**creatine**	**C00300**	27	28	27	28	26
**creatinine**	**C00791**	30	30	29	30	30
**CTP**	**C00063**	18	15	18	17	12
**cystathionine**	**C00542**	19	19	19	20	20
**cysteine**	**C00736-C00097**	16	15	19	21	19
**cysteine-S-sulfate**	**C05824**	27	27	22	23	23
**cystine**	**C01420**	23	23	27	28	27
**cytidine**	**C00475**	17	17	0	18	16
**cytosine**	**C00380**	20	20	19	19	20
**D-arabinono-1,4-lactone**	**C00652**	22	22	21	22	22
**D-erythrose-4-phosphate**	**C00279**	0	0	0	0	5
**dehydroascorbic acid**	**C05422**	0	0	20	21	20
**deoxyuridine**	**C00526**	20	19	17	19	18
**dihydroorotate**	**C00337**	17	17	21	20	21
**DL-DOPA**	**C00355**	18	17	18	17	18
**epinephrine**	**C00788**	19	19	18	19	19
**fumaric acid**	**C00122**	20	20	19	19	19
**GABA**	**C00334**	21	19	20	19	19
**GDP**	**C00035**	17	14	18	17	18
**glutamine**	**C00303**	27	27	29	29	29
**glutaminic acid**	**C00302**	28	28	24	25	24
**glutathione**	**C00051**	19	18	17	0	5
**glutathione disulfide**	**C00127**	21	20	21	20	20
**glyceric acid**	**C00258**	29	29	27	28	27
**glycine**	**C00037**	27	27	25	26	25
**glycylglycine**	**C02037**	0	18	0	0	0
**GMP**	**C00144**	13	13	15	18	16
**GTP**	**C00044**	17	13	14	13	5
**guanidine**	**C17349**	15	18	18	14	17
**guanidinoacetate**	**C00581**	22	23	20	21	21
**guanine**	**C00242**	15	18	0	0	4
**guanosine**	**C00387**	14	0	0	0	0
**histamine**	**C00388**	0	0	0	17	6
**histidine**	**C00768**	29	29	27	27	27
**homocysteine**	**C05330**	23	24	22	25	23
**hydroxyphenyllactic acid**	**C03672**	23	23	23	23	24
**hypoxanthine**	**C00262**	31	28	25	26	25
**IDP**	**C00104**	0	0	24	26	24
**imidazole-4-acetate**	**C02835**	23	23	22	22	22
**IMP**	**C00130**	13	0	23	24	22
**inosine**	**C00294**	18	0	0	19	5
**kynurenic acid**	**C01717**	19	19	19	19	20
**kynurenine**	**C01718**	25	25	25	26	25
**L-arginino-succinate**	**C03406**	19	20	18	19	19
**L-cysteic acid**	**C00506**	0	16	0	0	0
**L-NMMA**	**C03884**	26	26	25	26	26
**L-tyrosine methyl ester**	**C03404**	0	14	15	14	15
**lactic acid**	**C01432**	0	0	0	0	0
**levulinic acid**	**n/a**	0	0	22	0	0
**lysine**	**C00047/C00739**	31	31	30	31	30
**maleic acid**	**C01384**	0	17	16	0	0
**malic acid**	**C00711**	26	25	24	25	24
**methionine**	**C00073**	29	29	28	29	28
**methylglyoxal**	**C00546**	22	22	0	21	6
**methylmalonic acid**	**C02170**	25	25	24	27	26
**mevalonic acid**	**C00418**	22	23	22	23	22
**N-acetyl-DL-alanine**	**n/a**	25	26	25	26	26
**N-acetyl-L-glutamic acid**	**C00624**	17	0	0	0	0
**N-acetylaspartic acid**	**C01042**	0	0	17	18	0
**N-acetylaspartylglutamic acid**	**C12270**	17	18	19	20	18
**N-carbamoyl-L-aspartate**	**C00438**	18	17	0	0	0
**N-methyltryptamine**	**C06213**	0	0	15	16	15
**NAD+**	**C00003**	15	15	15	0	0
**NADP+**	**C00006**	0	0	0	13	5
**nicotinamide**	**C00153**	24	23	22	23	22
**NMN**	**C00455**	0	0	0	13	4
**octopamine**	**C04227**	17	17	20	21	20
**ornithine**	**C01602**	29	29	26	27	27
**orotic acid**	**C00295**	21	20	19	21	20
**pantothenic acid**	**C00864**	23	23	23	25	23
**phenylalanine**	**C02057**	32	32	30	30	30
**phosphocreatine**	**C02305**	20	19	21	20	20
**phosphoenolpyruvate**	**C00074**	0	0	19	17	19
**phosphorylcholine+**	**C00588**	24	23	23	23	23
**picolinic acid**	**C10164**	20	21	21	22	22
**proline**	**C16435**	32	32	32	32	33
**pseudouridine**	**C02067**	24	24	23	25	24
**putrescine**	**C00134**	27	20	17	17	15
**pyridoxine**	**C00314**	21	21	17	18	12
**quinolinic acid**	**C03722**	19	19	18	18	19
**S-adenosyl-L-methionine+**	**C00019**	18	14	20	20	20
**serine**	**C00716**	24	24	23	23	23
**spermidine**	**C00315**	25	25	25	25	25
**spermine**	**C00750**	24	24	24	24	24
**succinic acid**	**C00042**	25	24	22	22	22
**taurine**	**C00245**	29	30	27	29	28
**thiamine+**	**C00378**	0	14	17	19	6
**thymidine**	**C00214**	17	16	14	17	10
**thymine**	**C00178**	20	21	19	19	20
**tryptamine**	**C00398**	15	13	16	16	17
**tryptophan**	**C00806**	30	30	30	30	29
**tyramine**	**C00483**	19	20	19	19	19
**tyrosine**	**C01536**	30	30	30	30	29
**UDP**	**C00015**	13	0	14	15	11
**uracil**	**C00106**	23	22	20	21	14
**uric acid**	**C00366**	29	30	29	30	30
**uridine**	**C00299**	25	25	23	25	24
**UTP**	**C00075**	13	0	15	0	6
**valine**	**C16436**	30	30	30	31	30
**xanthine**	**C00385**	26	25	22	23	22
**xanthosine**	**C01762**	17	0	14	13	15
**xanthurenic acid**	**C02470**	17	16	19	18	19
